# Editorial: Molecular mechanisms in ocular development and disease

**DOI:** 10.3389/fcell.2023.1244123

**Published:** 2023-06-29

**Authors:** Rajalekshmy Shyam, Daisy Y. Shu, Elizabeth Zuniga-Sanchez, Deepika Vasudevan

**Affiliations:** ^1^ Indiana University School of Optometry, Bloomington, IN, United States; ^2^ Schepens Eye Research Institute of Massachusetts Eye and Ear, Boston, MA, United States; ^3^ Department of Ophthalmology, Harvard Medical School, Boston, MA, United States; ^4^ Department of Ophthalmology, Baylor College of Medicine, Houston, TX, United States; ^5^ Department of Cell Biology, University of Pittsburgh School of Medicine, Pittsburgh, PA, United States

**Keywords:** eye developent, retina, cornea, ophthalmological disease, optic nerve, photoreceptor

## Introduction

From developmental defects that affect infants and children to age-related defects that impact older adults, a vast and complex range of ophthalmological disorders affects nearly 2.2 billion individuals globally. Each step in light perception and processing involves multiple cell types and signaling pathways that we collectively refer to as the visual system. A substantial portion of our understanding of this remarkable system comes from basic research using model organisms such as mice, rats, zebrafish and fruit flies. Such research has been crucial not only in identifying signaling pathways that instruct normal development and functioning of the eye, but also in tracing the etiology of ocular pathologies. This Research Topic presents a collection of new research and comprehensive review articles that describe recent advancements in our understanding of visual development and ocular diseases.

The articles in this Research Topic can be broadly classified into three groups: a set of review articles summarizing recent progress in specific disorders, a set of research articles focused on disease etiology and interventions, and a third set of research articles focused on the development of the visual system.

The first of the review articles by He et al. provides a comprehensive overview of technological advances in gene therapy as a promising approach to treating glaucoma, which is the leading cause of blindness worldwide. The current mainstay for treating glaucoma is through topical medications or surgery to lower the intraocular pressure (IOP). However, there are many risk factors outside of elevated IOP that drive glaucoma, which call for new gene therapy-based strategies targeting pathogenic genes that have been linked to primary open angle glaucoma (MYOC, NTF4, OPTN, WDR36).

Next, Wei et al. review the multifactorial mechanisms underlying diabetic retinopathy including oxidative stress, inflammation, neovascularization and neurodegeneration. They extensively discuss the key role of the “gut-retinal axis” in maintaining normal metabolic function and immune regulation in the retina. This is critical because hyperglycemia in diabetic patients can lead to dysregulation of the gut microbiota, resulting in intestinal inflammation and changes in the metabolome. The authors also highlight key areas of research that will inform the development of therapeutic strategies that prevent disease progression while treating patient symptoms. Krueger and Morris outline the unanswered questions on the pathogenesis of CHARGE syndrome, a genetic disorder characterized by ocular coloboma, heart defects, choanal atresia, growth retardation, genital abnormalities, and ear abnormalities. The authors place special emphasis on pathogenic variations in the protein Chromodomain helicase DNA binding protein 7 (CHD7), which causes the majority of CHARGE syndrome cases. Consistent with the theme of this Research Topic, the article discusses known molecular roles for CHD7 and its effects on vertebrate ocular development and function. Finally, an article by Chen et al. discusses the need to study the role of lipid metabolism, as effected by peroxisomes, in eye diseases. Notably, the authors summarize cases of ocular symptoms in patients with peroxisomal disorders. Peroxisomes are highly specialized enzyme-rich organelles that mediate degradation of long-chain fatty acids amongst other substrates, and communicate extensively with other organelles critical for retinal function such as the endoplasmic reticulum, mitochondria and lysosomes.

In the cluster of research articles focused on disease etiology and treatment strategies, Tribble et al. propose a new pharmacological approach using valproic acid as a neuroprotective agent for retinal ganglion cells. Treatment with valproic acid improved microglial architecture and prevented astrocyte remodeling in the retina, indicating the potential of this treatment modality for ocular neurodegenerative diseases such as glaucoma. Next, Minogue et al. examined the protein composition of the lens in two mouse models that develop cataracts due to mutations in connexin46 and connexin50. They use micro-computed tomography to show that there is calcium phosphate present in the form of apatite in the lens of mutant mice but not in the wildtype. This work supports the idea that cataracts form due to abnormal mineralization within the lens. Another cutting edge study by Tan et al. team utilize live cell imaging to analyze mitochondrial dynamics and functions in Retinal Pigment Epithelial (RPE) cells. Strikingly, they show that typical lab fixation protocols disrupt mitochondria integrity, thus highlighting the suitability of live-cell imaging for studying mitochondrial dynamics in RPE cells. Their results find substantial loss in mitochondrial integrity in albino mice compared to pigmented mice, suggesting critical roles for pigmentation in cellular metabolism. This cluster was home to another methods paper by Parreno et al. who provide detailed experimental protocols to investigate the molecular expression patterns and cellular structures in lens epithelial cells. The lens of the eye is covered by a thin layer of epithelial cells. One of the complications associated with cataract surgery is that the lens epithelial cells undergo an epithelial-mesenchymal transition resulting in the formation of a secondary cataract. Their protocols span multiple lens epithelia systems including native, primary culture, and immortalized cells, making this an excellent resource for studying lens cell biology.

The third set of research articles showcased mouse and *Drosophila* models to study the development of the visual system. Patel et al. used a compound mouse knockout model to show that the bZIP transcription factors, MafG and MafK are required for lens development. They dissect the underlying mechanism using transcriptomics to reveal clusters of target genes associated with cytoskeletal and extracellular matrix functions that likely mediate the effects of MafG and MafK in lens development. Fuhrmann et al. research group similarly uses a mouse model to investigate the role of Porcn, a regulator of the Wnt signaling pathway during early stages of ocular development. Dysregulation of signaling cascades that regulate the morphogenetic events during early development results in congenital ocular malformations such as microphthalmia, anophthalmia, and coloboma. This work finds that temporal loss of Porcn in ocular tissues during development acutely recapitulates severe microphthalmia seen in Focal Dermal Hypoplasia. Importantly, their work sets the stage for future studies to identify the specific cell types where Porcn is required for optic cup development. This section was also home to such advanced studies on cell type specific gene regulation such as Bunker et al., who pinpoint a role for the evolutionarily conserved transcription complexes Blimp-1 and Hr3 in blue-light sensitive photoreceptor specification. In the case of the *Drosophila* eye, as with the vertebrate eye, the proportion of blue- and -green-light sensitive photoreceptors is determined by seemingly stochastic expression of transcription factors. The authors use elegant *Drosophila* genetics to show that Blimp-1 and Hr3 act as repressors of wrts, a well-known modulator of photoreceptor specification, to stochastically designate blue light-sensitive photoreceptors. This original research work is perfectly complemented by a review where Warren and Kumar artfully discuss two contrasting models for how the *Drosophila* retina is patterned: the first model makes a case for morphogen-driven gradients across stationary cells, and the second argues that mechanically driven cell flow contributes to retinal pattern formation.

## Summary

The research findings and proposed questions emanating from this Research Topic are highly relevant to understanding the complexity that underlies development, function, and diseases in the visual system. In a field that has spanned decades yet continues to pursue fundamentally formative problems, Research Topic articles such as this one hopefully serves as an influential frame of a reference within the coordinates of basic vision science.

